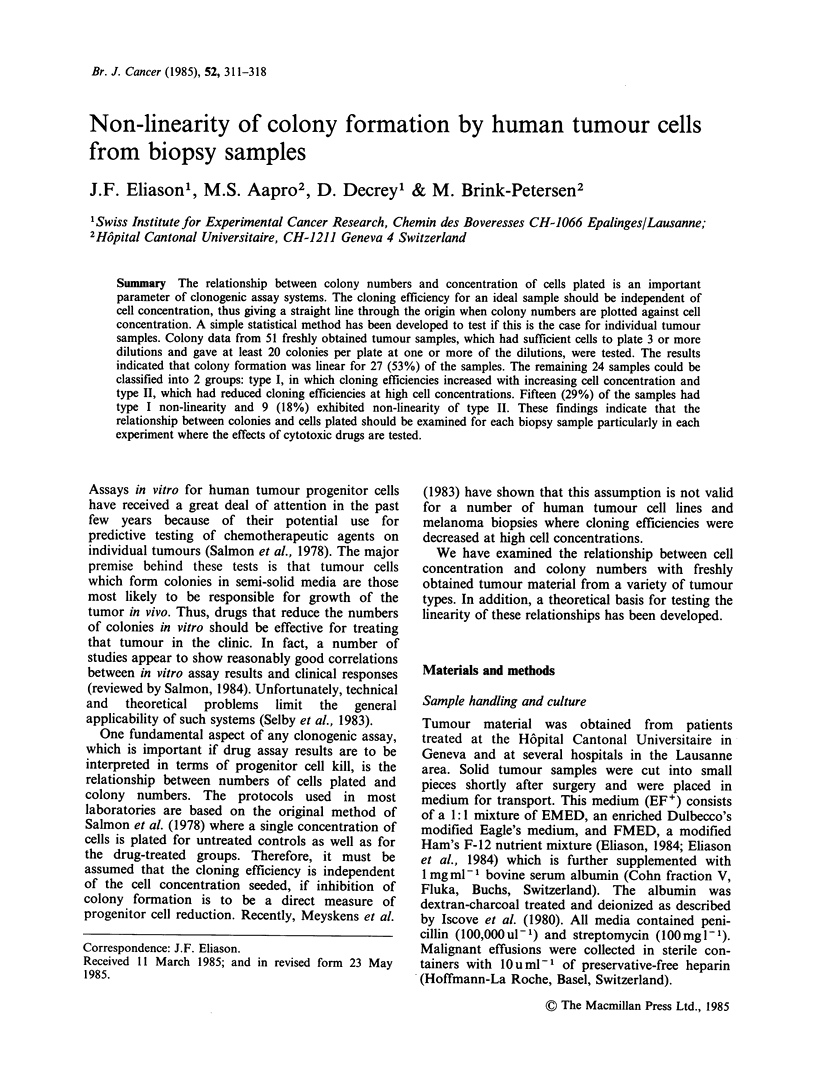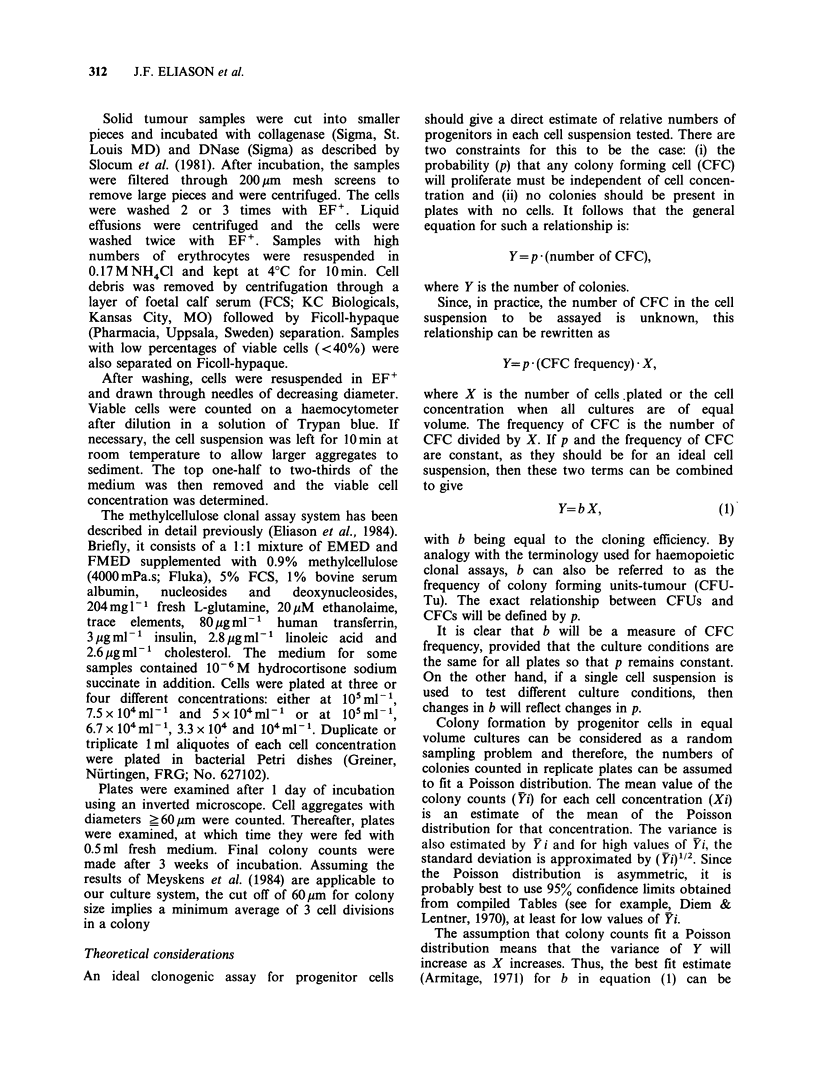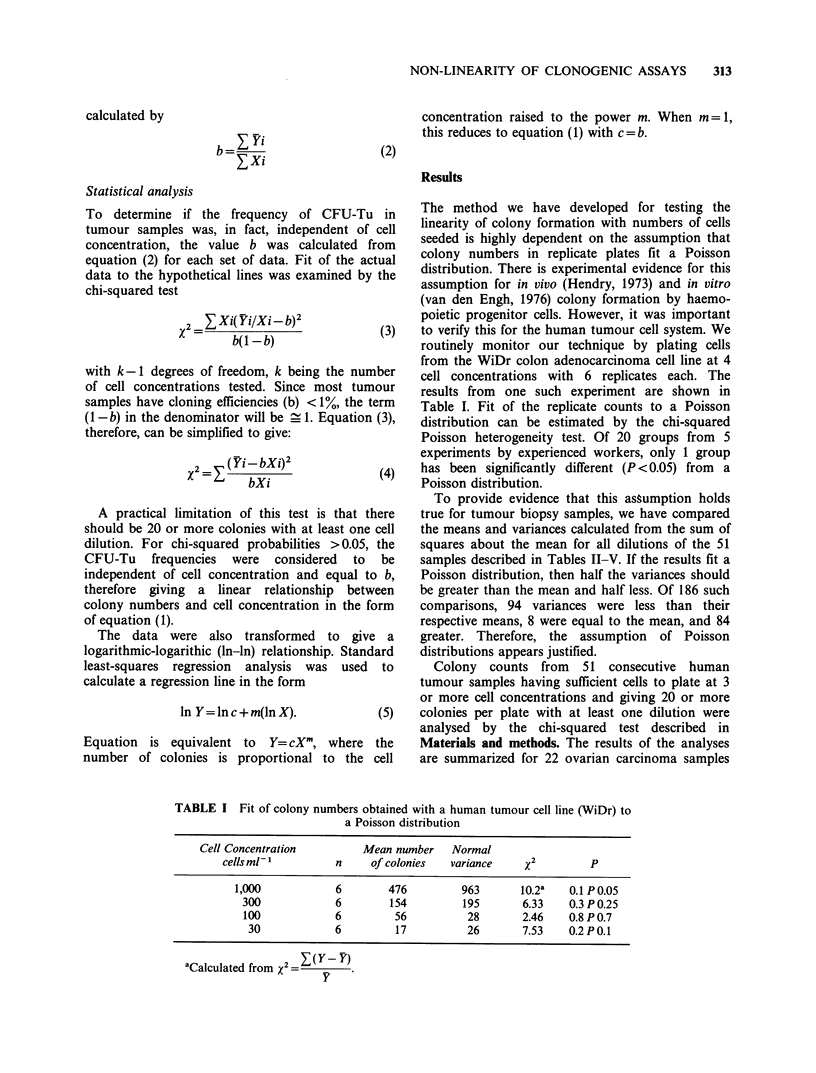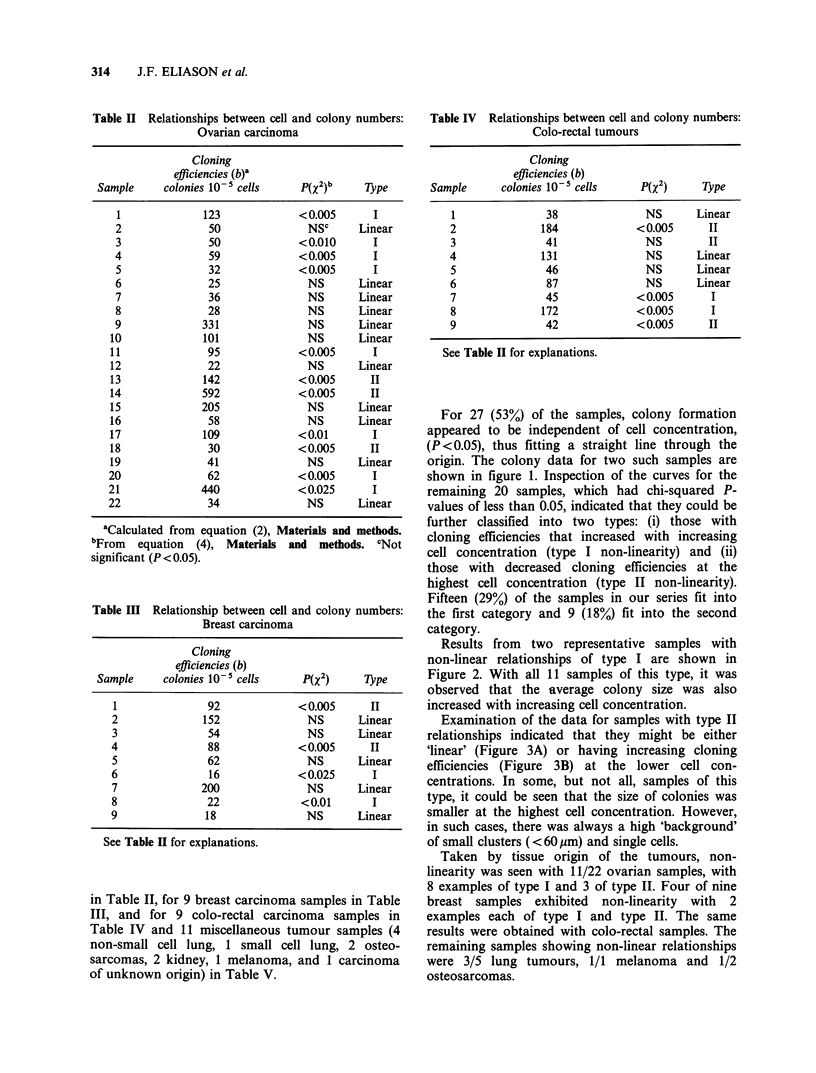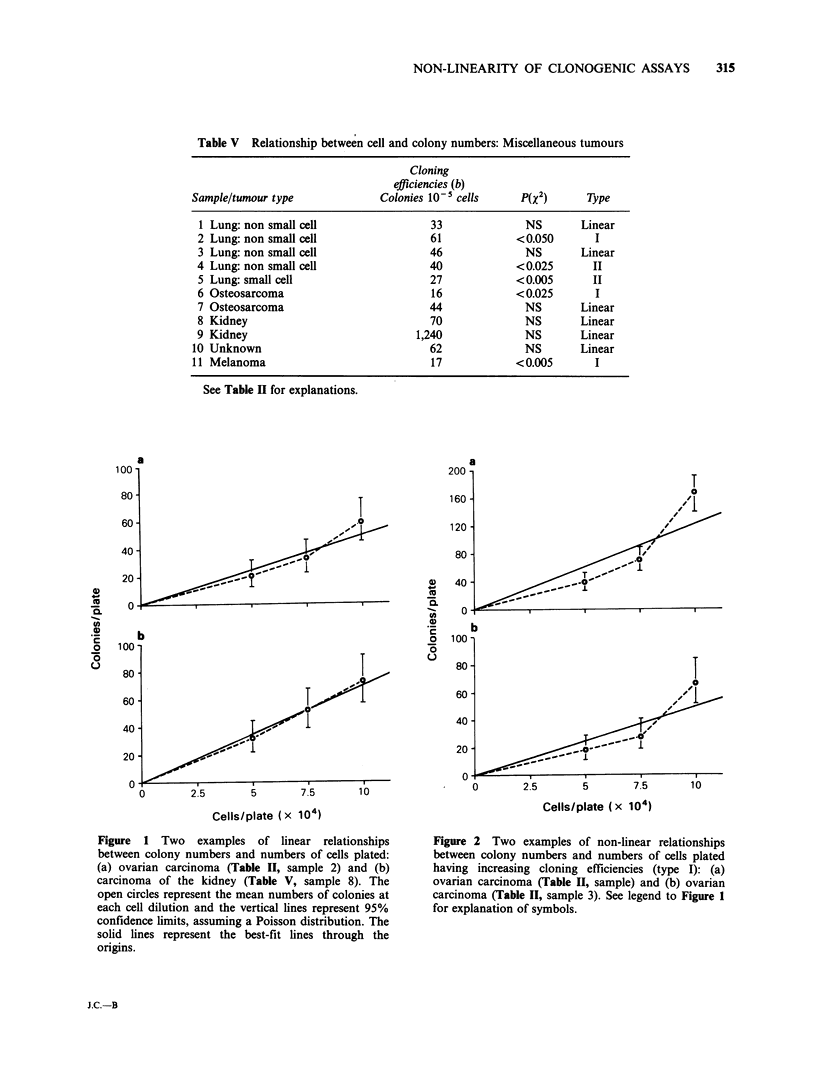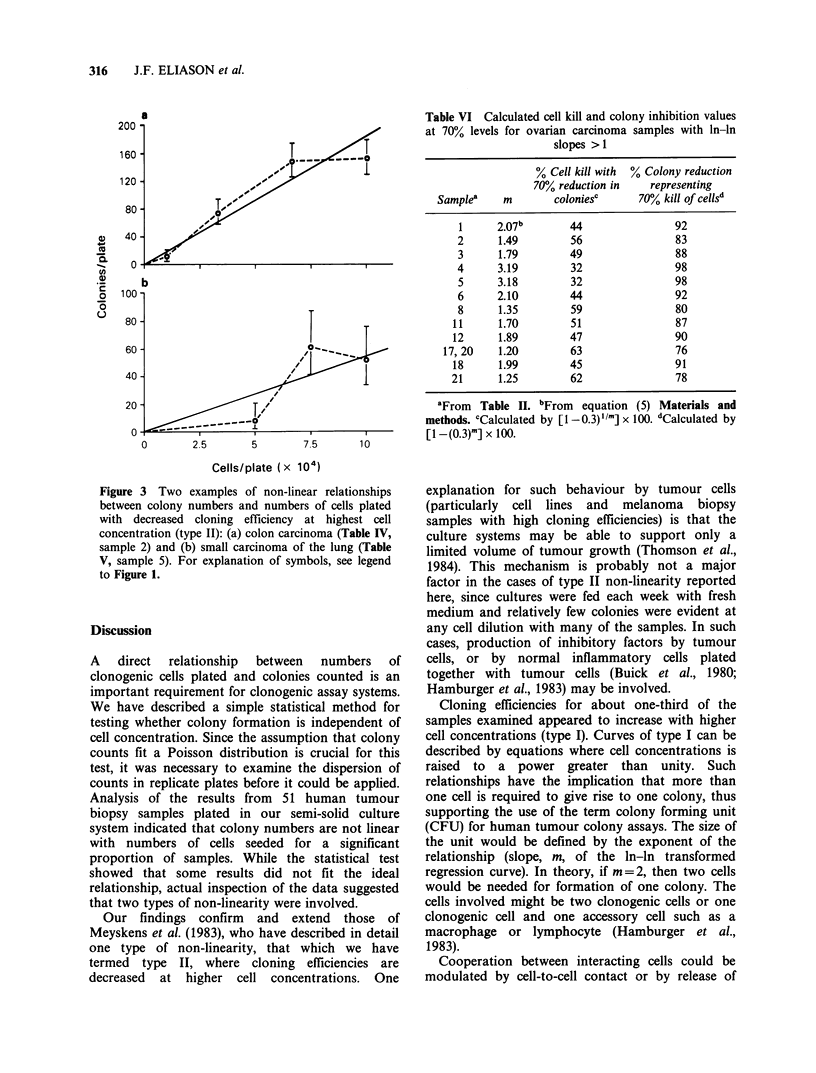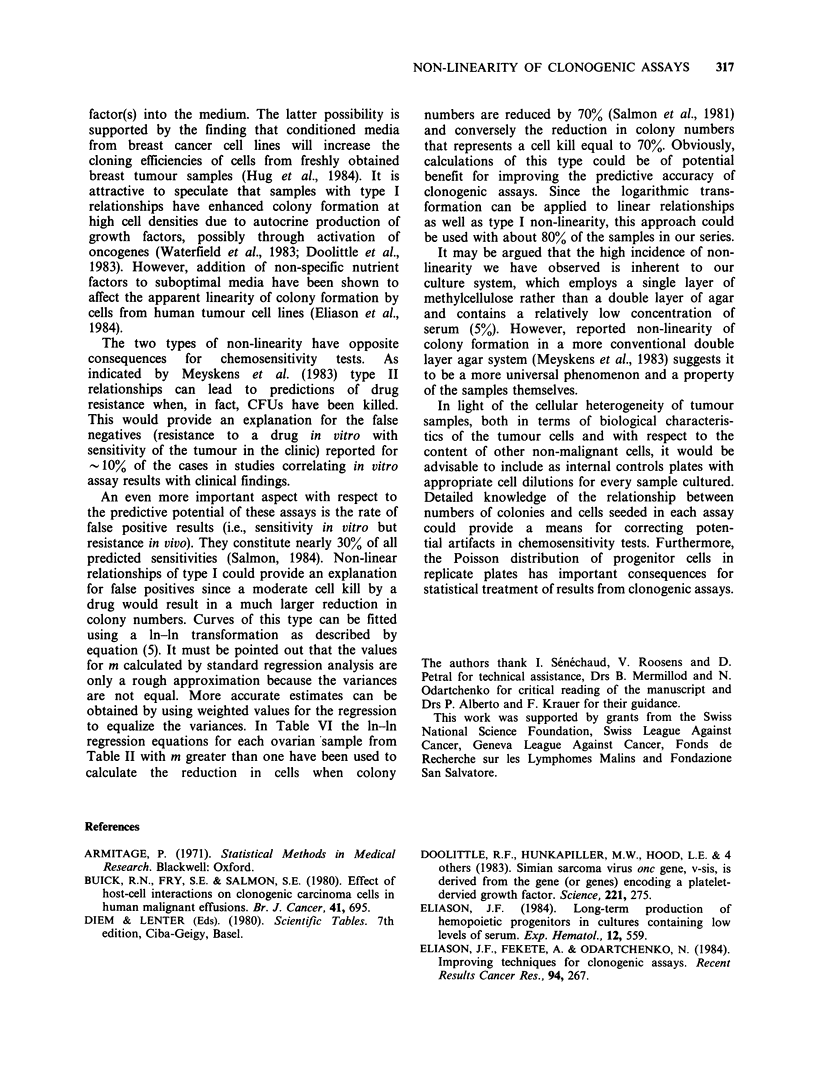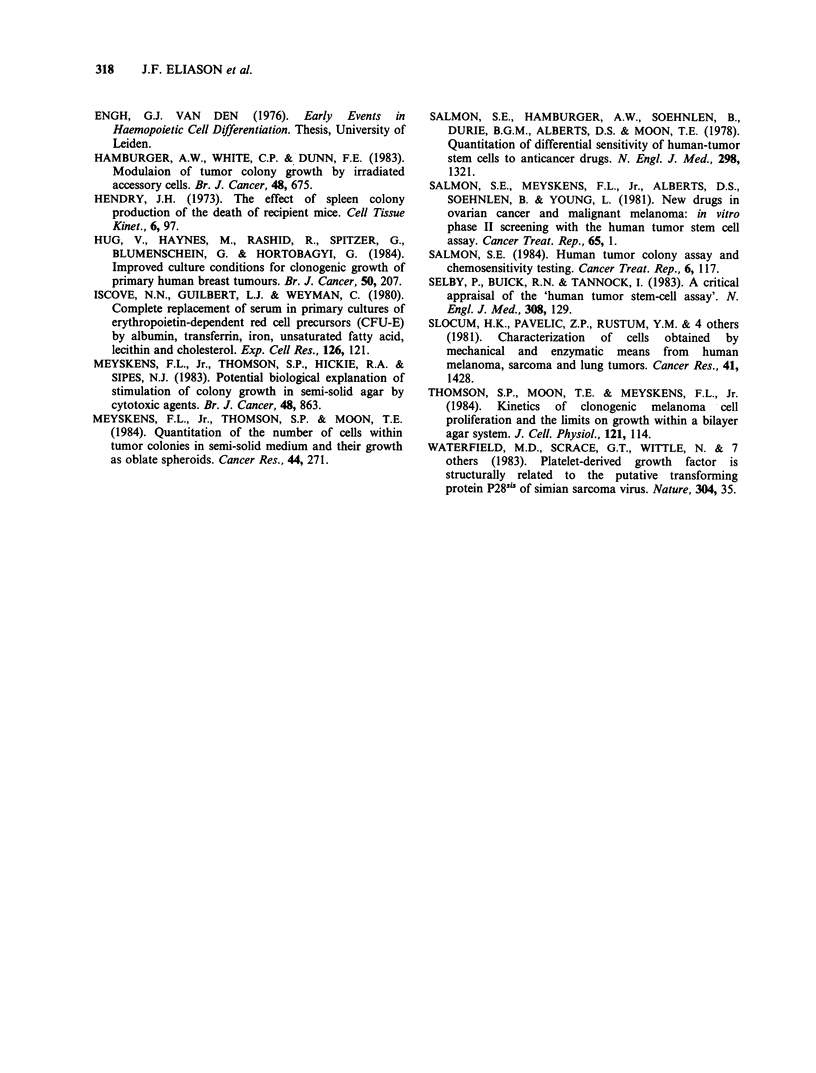# Non-linearity of colony formation by human tumour cells from biopsy samples.

**DOI:** 10.1038/bjc.1985.195

**Published:** 1985-09

**Authors:** J. F. Eliason, M. S. Aapro, D. Decrey, M. Brink-Petersen

## Abstract

The relationship between colony numbers and concentration of cells plated is an important parameter of clonogenic assay systems. The cloning efficiency for an ideal sample should be independent of cell concentration, thus giving a straight line through the origin when colony numbers are plotted against cell concentration. A simple statistical method has been developed to test if this is the case for individual tumour samples. Colony data from 51 freshly obtained tumour samples, which had sufficient cells to plate 3 or more dilutions and gave at least 20 colonies per plate at one or more of the dilutions, were tested. The results indicated that colony formation was linear for 27 (53%) of the samples. The remaining 24 samples could be classified into 2 groups: type I, in which cloning efficiencies increased with increasing cell concentration and type II, which had reduced cloning efficiencies at high cell concentrations. Fifteen (29%) of the samples had type I non-linearity and 9 (18%) exhibited non-linearity of type II. These findings indicate that the relationship between colonies and cells plated should be examined for each biopsy sample particularly in each experiment where the effects of cytotoxic drugs are tested.


*Br. J. Cancer* (1985), **52**, 311–318

### Non-linearity of colony formation by human tumour cells from biopsy samples

J.F. Eliason[1], M.S. Aapro[2], D. Decrey[1] & M. Brink-Petersen[2]

[1]*Swiss Institute for Experimental Cancer Research, Chemin des Boveresses CH-1066 Epalinges/Lausanne;*
[2]*Hôpital Cantonal Universitaire, CH-1211 Geneva 4 Switzerland*

**Summary** The relationship between colony numbers and concentration of cells plated is an important parameter of clonogenic assay systems. The cloning efficiency for an ideal sample should be independent of cell concentration, thus giving a straight line through the origin when colony numbers are plotted against cell concentration. A simple statistical method has been developed to test if this is the case for individual tumour samples. Colony data from 51 freshly obtained tumour samples, which had sufficient cells to plate 3 or more dilutions and gave at least 20 colonies per plate at one or more of the dilutions, were tested. The results indicated that colony formation was linear for 27 (53%) of the samples. The remaining 24 samples could be classified into 2 groups: type I, in which cloning efficiencies increased with increasing cell concentration and type II, which had reduced cloning efficiencies at high cell concentrations. Fifteen (29%) of the samples had type I non-linearity and 9 (18%) exhibited non-linearity of type II. These findings indicate that the relationship between colonies and cells plated should be examined for each biopsy sample particularly in each experiment where the effects of cytotoxic drugs are tested.

Assays *in vitro* for human tumour progenitor cells have received a great deal of attention in the past few years because of their potential use for predictive testing of chemotherapeutic agents on individual tumours (Salmon *et al.*, 1978). The major premise behind these tests is that tumour cells which form colonies in semi-solid media are those most likely to be responsible for growth of the tumor *in vivo*. Thus, drugs that reduce the numbers of colonies *in vitro* should be effective for treating that tumour in the clinic. In fact, a number of studies appear to show reasonably good correlations between *in vitro* assay results and clinical responses (reviewed by Salmon, 1984). Unfortunately, technical and theoretical problems limit the general applicability of such systems (Selby *et al.*, 1983).

One fundamental aspect of any clonogenic assay, which is important if drug assay results are to be interpreted in terms of progenitor cell kill, is the relationship between numbers of cells plated and colony numbers. The protocols used in most laboratories are based on the original method of Salmon *et al.* (1978) where a single concentration of cells is plated for untreated controls as well as for the drug-treated groups. Therefore, it must be assumed that the cloning efficiency is independent of the cell concentration seeded, if inhibition of colony formation is to be a direct measure of progenitor cell reduction. Recently, Meyskens *et al.* (1983) have shown that this assumption is not valid for a number of human tumour cell lines and melanoma biopsies where cloning efficiencies were decreased at high cell concentrations.

We have examined the relationship between cell concentration and colony numbers with freshly obtained tumour material from a variety of tumour types. In addition, a theoretical basis for testing the linearity of these relationships has been developed.

Correspondence: J.F. Eliason.
Received 11 March 1985; and in revised form 23 May 1985.

### Materials and methods

### *Sample handling and culture*

Tumour material was obtained from patients treated at the Hôpital Cantonal Universitaire in Geneva and at several hospitals in the Lausanne area. Solid tumour samples were cut into small pieces shortly after surgery and were placed in medium for transport. This medium ($EF^+$) consists of a 1:1 mixture of EMED, an enriched Dulbecco's modified Eagle's medium, and FMED, a modified Ham's F-12 nutrient mixture (Eliason, 1984; Eliason *et al.*, 1984) which is further supplemented with $1\,mg\,ml^{-1}$ bovine serum albumin (Cohn fraction V, Fluka, Buchs, Switzerland). The albumin was dextran-charcoal treated and deionized as described by Iscove *et al.* (1980). All media contained penicillin ($100{,}000\,u\,l^{-1}$) and streptomycin ($100\,mg\,l^{-1}$). Malignant effusions were collected in sterile containers with $10\,u\,ml^{-1}$ of preservative-free heparin (Hoffmann-La Roche, Basel, Switzerland).

© The Macmillan Press Ltd., 1985

Solid tumour samples were cut into smaller pieces and incubated with collagenase (Sigma, St. Louis MD) and DNase (Sigma) as described by Slocum *et al.* (1981). After incubation, the samples were filtered through 200 $\mu$m mesh screens to remove large pieces and were centrifuged. The cells were washed 2 or 3 times with $EF^+$. Liquid effusions were centrifuged and the cells were washed twice with $EF^+$. Samples with high numbers of erythrocytes were resuspended in 0.17 M $NH_4Cl$ and kept at 4°C for 10 min. Cell debris was removed by centrifugation through a layer of foetal calf serum (FCS; KC Biologicals, Kansas City, MO) followed by Ficoll-hypaque (Pharmacia, Uppsala, Sweden) separation. Samples with low percentages of viable cells (<40%) were also separated on Ficoll-hypaque.

After washing, cells were resuspended in $EF^+$ and drawn through needles of decreasing diameter. Viable cells were counted on a haemocytometer after dilution in a solution of Trypan blue. If necessary, the cell suspension was left for 10 min at room temperature to allow larger aggregates to sediment. The top one-half to two-thirds of the medium was then removed and the viable cell concentration was determined.

The methylcellulose clonal assay system has been described in detail previously (Eliason *et al.*, 1984). Briefly, it consists of a 1:1 mixture of EMED and FMED supplemented with 0.9% methylcellulose (4000 mPa.s; Fluka), 5% FCS, 1% bovine serum albumin, nucleosides and deoxynucleosides, 204 mg $l^{-1}$ fresh L-glutamine, 20 $\mu$M ethanolaime, trace elements, 80 $\mu g\,ml^{-1}$ human transferrin, 3 $\mu g\,ml^{-1}$ insulin, 2.8 $\mu g\,ml^{-1}$ linoleic acid and 2.6 $\mu g\,ml^{-1}$ cholesterol. The medium for some samples contained $10^{-6}$ M hydrocortisone sodium succinate in addition. Cells were plated at three or four different concentrations: either at $10^5\,ml^{-1}$, $7.5\times10^4\,ml^{-1}$ and $5\times10^4\,ml^{-1}$ or at $10^5\,ml^{-1}$, $6.7\times10^4\,ml^{-1}$, $3.3\times10^4$ and $10^4\,ml^{-1}$. Duplicate or triplicate 1 ml aliquotes of each cell concentration were plated in bacterial Petri dishes (Greiner, Nürtingen, FRG; No. 627102).

Plates were examined after 1 day of incubation using an inverted microscope. Cell aggregates with diameters $\geqq 60\,\mu m$ were counted. Thereafter, plates were examined, at which time they were fed with 0.5 ml fresh medium. Final colony counts were made after 3 weeks of incubation. Assuming the results of Meyskens *et al.* (1984) are applicable to our culture system, the cut off of 60 $\mu$m for colony size implies a minimum average of 3 cell divisions in a colony

*Theoretical considerations*

An ideal clonogenic assay for progenitor cells should give a direct estimate of relative numbers of progenitors in each cell suspension tested. There are two constraints for this to be the case: (i) the probability ($p$) that any colony forming cell (CFC) will proliferate must be independent of cell concentration and (ii) no colonies should be present in plates with no cells. It follows that the general equation for such a relationship is:

$$Y = p \cdot (\text{number of CFC}),$$

where $Y$ is the number of colonies.

Since, in practice, the number of CFC in the cell suspension to be assayed is unknown, this relationship can be rewritten as

$$Y = p \cdot (\text{CFC frequency}) \cdot X,$$

where $X$ is the number of cells plated or the cell concentration when all cultures are of equal volume. The frequency of CFC is the number of CFC divided by $X$. If $p$ and the frequency of CFC are constant, as they should be for an ideal cell suspension, then these two terms can be combined to give

$$Y = bX, \quad (1)$$

with $b$ being equal to the cloning efficiency. By analogy with the terminology used for haemopoietic clonal assays, $b$ can also be referred to as the frequency of colony forming units-tumour (CFU-Tu). The exact relationship between CFUs and CFCs will be defined by $p$.

It is clear that $b$ will be a measure of CFC frequency, provided that the culture conditions are the same for all plates so that $p$ remains constant. On the other hand, if a single cell suspension is used to test different culture conditions, then changes in $b$ will reflect changes in $p$.

Colony formation by progenitor cells in equal volume cultures can be considered as a random sampling problem and therefore, the numbers of colonies counted in replicate plates can be assumed to fit a Poisson distribution. The mean value of the colony counts ($\bar{Y}i$) for each cell concentration ($Xi$) is an estimate of the mean of the Poisson distribution for that concentration. The variance is also estimated by $\bar{Y}i$ and for high values of $\bar{Y}i$, the standard deviation is approximated by $(\bar{Y}i)^{1/2}$. Since the Poisson distribution is asymmetric, it is probably best to use 95% confidence limits obtained from compiled Tables (see for example, Diem & Lentner, 1970), at least for low values of $\bar{Y}i$.

The assumption that colony counts fit a Poisson distribution means that the variance of $Y$ will increase as $X$ increases. Thus, the best fit estimate (Armitage, 1971) for $b$ in equation (1) can be calculated by

$$b=\frac{\sum \bar{Y}i}{\sum Xi} \quad (2)$$

*Statistical analysis*

To determine if the frequency of CFU-Tu in tumour samples was, in fact, independent of cell concentration, the value $b$ was calculated from equation (2) for each set of data. Fit of the actual data to the hypothetical lines was examined by the chi-squared test

$$\chi^2=\frac{\sum Xi(\bar{Y}i/Xi-b)^2}{b(1-b)} \quad (3)$$

with $k-1$ degrees of freedom, $k$ being the number of cell concentrations tested. Since most tumour samples have cloning efficiencies (b) $<1\%$, the term $(1-b)$ in the denominator will be $\cong 1$. Equation (3), therefore, can be simplified to give:

$$\chi^2=\sum\frac{(\bar{Y}i-bXi)^2}{bXi} \quad (4)$$

A practical limitation of this test is that there should be 20 or more colonies with at least one cell dilution. For chi-squared probabilities $>0.05$, the CFU-Tu frequencies were considered to be independent of cell concentration and equal to $b$, therefore giving a linear relationship between colony numbers and cell concentration in the form of equation (1).

The data were also transformed to give a logarithmic-logarithic (ln–ln) relationship. Standard least-squares regression analysis was used to calculate a regression line in the form

$$\ln Y=\ln c+m(\ln X). \quad (5)$$

Equation is equivalent to $Y=cX^m$, where the number of colonies is proportional to the cell concentration raised to the power $m$. When $m=1$, this reduces to equation (1) with $c=b$.

### Results

The method we have developed for testing the linearity of colony formation with numbers of cells seeded is highly dependent on the assumption that colony numbers in replicate plates fit a Poisson distribution. There is experimental evidence for this assumption for *in vivo* (Hendry, 1973) and *in vitro* (van den Engh, 1976) colony formation by haemopoietic progenitor cells. However, it was important to verify this for the human tumour cell system. We routinely monitor our technique by plating cells from the WiDr colon adenocarcinoma cell line at 4 cell concentrations with 6 replicates each. The results from one such experiment are shown in Table I. Fit of the replicate counts to a Poisson distribution can be estimated by the chi-squared Poisson heterogeneity test. Of 20 groups from 5 experiments by experienced workers, only 1 group has been significantly different ($P<0.05$) from a Poisson distribution.

To provide evidence that this assumption holds true for tumour biopsy samples, we have compared the means and variances calculated from the sum of squares about the mean for all dilutions of the 51 samples described in Tables II–V. If the results fit a Poisson distribution, then half the variances should be greater than the mean and half less. Of 186 such comparisons, 94 variances were less than their respective means, 8 were equal to the mean, and 84 greater. Therefore, the assumption of Poisson distributions appears justified.

Colony counts from 51 consecutive human tumour samples having sufficient cells to plate at 3 or more cell concentrations and giving 20 or more colonies per plate with at least one dilution were analysed by the chi-squared test described in **Materials and methods.** The results of the analyses are summarized for 22 ovarian carcinoma samples

**TABLE I** Fit of colony numbers obtained with a human tumour cell line (WiDr) to a Poisson distribution

| *Cell Concentration cells ml*$^{-1}$ | *n* | *Mean number of colonies* | *Normal variance* | $\chi^2$ | *P* |
|---|---|---|---|---|---|
| 1,000 | 6 | 476 | 963 | 10.2[a] | 0.1 *P* 0.05 |
| 300 | 6 | 154 | 195 | 6.33 | 0.3 *P* 0.25 |
| 100 | 6 | 56 | 28 | 2.46 | 0.8 *P* 0.7 |
| 30 | 6 | 17 | 26 | 7.53 | 0.2 *P* 0.1 |

[a]Calculated from $\chi^2=\frac{\sum(Y-\bar{Y})}{\bar{Y}}$.

**Table II** Relationships between cell and colony numbers: Ovarian carcinoma

| *Sample* | *Cloning efficiencies (b)*[a] *colonies* $10^{-5}$ *cells* | $P(\chi^2)$[b] | *Type* |
|---|---|---|---|
| 1 | 123 | <0.005 | I |
| 2 | 50 | NS[c] | Linear |
| 3 | 50 | <0.010 | I |
| 4 | 59 | <0.005 | I |
| 5 | 32 | <0.005 | I |
| 6 | 25 | NS | Linear |
| 7 | 36 | NS | Linear |
| 8 | 28 | NS | Linear |
| 9 | 331 | NS | Linear |
| 10 | 101 | NS | Linear |
| 11 | 95 | <0.005 | I |
| 12 | 22 | NS | Linear |
| 13 | 142 | <0.005 | II |
| 14 | 592 | <0.005 | II |
| 15 | 205 | NS | Linear |
| 16 | 58 | NS | Linear |
| 17 | 109 | <0.01 | I |
| 18 | 30 | <0.005 | II |
| 19 | 41 | NS | Linear |
| 20 | 62 | <0.005 | I |
| 21 | 440 | <0.025 | I |
| 22 | 34 | NS | Linear |

[a]Calculated from equation (2), **Materials and methods.** [b]From equation (4), **Materials and methods.** [c]Not significant ($P<0.05$).

**Table III** Relationship between cell and colony numbers: Breast carcinoma

| *Sample* | *Cloning efficiencies (b) colonies* $10^{-5}$ *cells* | $P(\chi^2)$ | *Type* |
|---|---|---|---|
| 1 | 92 | <0.005 | II |
| 2 | 152 | NS | Linear |
| 3 | 54 | NS | Linear |
| 4 | 88 | <0.005 | II |
| 5 | 62 | NS | Linear |
| 6 | 16 | <0.025 | I |
| 7 | 200 | NS | Linear |
| 8 | 22 | <0.01 | I |
| 9 | 18 | NS | Linear |

See **Table II** for explanations.

in Table II, for 9 breast carcinoma samples in Table III, and for 9 colo-rectal carcinoma samples in Table IV and 11 miscellaneous tumour samples (4 non-small cell lung, 1 small cell lung, 2 osteosarcomas, 2 kidney, 1 melanoma, and 1 carcinoma of unknown origin) in Table V.

**Table IV** Relationships between cell and colony numbers: Colo-rectal tumours

| *Sample* | *Cloning efficiencies (b) colonies* $10^{-5}$ *cells* | $P(\chi^2)$ | *Type* |
|---|---|---|---|
| 1 | 38 | NS | Linear |
| 2 | 184 | <0.005 | II |
| 3 | 41 | NS | II |
| 4 | 131 | NS | Linear |
| 5 | 46 | NS | Linear |
| 6 | 87 | NS | Linear |
| 7 | 45 | <0.005 | I |
| 8 | 172 | <0.005 | I |
| 9 | 42 | <0.005 | II |

See **Table II** for explanations.

For 27 (53%) of the samples, colony formation appeared to be independent of cell concentration, ($P<0.05$), thus fitting a straight line through the origin. The colony data for two such samples are shown in figure 1. Inspection of the curves for the remaining 20 samples, which had chi-squared *P*-values of less than 0.05, indicated that they could be further classified into two types: (i) those with cloning efficiencies that increased with increasing cell concentration (type I non-linearity) and (ii) those with decreased cloning efficiencies at the highest cell concentration (type II non-linearity). Fifteen (29%) of the samples in our series fit into the first category and 9 (18%) fit into the second category.

Results from two representative samples with non-linear relationships of type I are shown in Figure 2. With all 11 samples of this type, it was observed that the average colony size was also increased with increasing cell concentration.

Examination of the data for samples with type II relationships indicated that they might be either 'linear' (Figure 3A) or having increasing cloning efficiencies (Figure 3B) at the lower cell concentrations. In some, but not all, samples of this type, it could be seen that the size of colonies was smaller at the highest cell concentration. However, in such cases, there was always a high 'background' of small clusters ($<60\,\mu m$) and single cells.

Taken by tissue origin of the tumours, non-linearity was seen with 11/22 ovarian samples, with 8 examples of type I and 3 of type II. Four of nine breast samples exhibited non-linearity with 2 examples each of type I and type II. The same results were obtained with colo-rectal samples. The remaining samples showing non-linear relationships were 3/5 lung tumours, 1/1 melanoma and 1/2 osteosarcomas.

**Table V** Relationship between cell and colony numbers: Miscellaneous tumours

| *Sample/tumour type* | *Cloning efficiencies (b) Colonies $10^{-5}$ cells* | $P(\chi^2)$ | *Type* |
|---|---|---|---|
| 1 Lung: non small cell | 33 | NS | Linear |
| 2 Lung: non small cell | 61 | <0.050 | I |
| 3 Lung: non small cell | 46 | NS | Linear |
| 4 Lung: non small cell | 40 | <0.025 | II |
| 5 Lung: small cell | 27 | <0.005 | II |
| 6 Osteosarcoma | 16 | <0.025 | I |
| 7 Osteosarcoma | 44 | NS | Linear |
| 8 Kidney | 70 | NS | Linear |
| 9 Kidney | 1,240 | NS | Linear |
| 10 Unknown | 62 | NS | Linear |
| 11 Melanoma | 17 | <0.005 | I |

See **Table II** for explanations.

**Figure 1** Two examples of linear relationships between colony numbers and numbers of cells plated: (a) ovarian carcinoma (**Table II**, sample 2) and (b) carcinoma of the kidney (**Table V**, sample 8). The open circles represent the mean numbers of colonies at each cell dilution and the vertical lines represent 95% confidence limits, assuming a Poisson distribution. The solid lines represent the best-fit lines through the origins.

**Figure 2** Two examples of non-linear relationships between colony numbers and numbers of cells plated having increasing cloning efficiencies (type I): (a) ovarian carcinoma (**Table II**, sample) and (b) ovarian carcinoma (**Table II**, sample 3). See legend to **Figure 1** for explanation of symbols.

**Figure 3** Two examples of non-linear relationships between colony numbers and numbers of cells plated with decreased cloning efficiency at highest cell concentration (type II): (a) colon carcinoma (**Table IV**, sample 2) and (b) small carcinoma of the lung (**Table V**, sample 5). For explanation of symbols, see legend to **Figure 1.**

**Table VI** Calculated cell kill and colony inhibition values at 70% levels for ovarian carcinoma samples with ln–ln slopes >1

| *Sample*[a] | *m* | *% Cell kill with 70% reduction in colonies*[c] | *% Colony reduction representing 70% kill of cells*[d] |
|---|---|---|---|
| 1 | 2.07[b] | 44 | 92 |
| 2 | 1.49 | 56 | 83 |
| 3 | 1.79 | 49 | 88 |
| 4 | 3.19 | 32 | 98 |
| 5 | 3.18 | 32 | 98 |
| 6 | 2.10 | 44 | 92 |
| 8 | 1.35 | 59 | 80 |
| 11 | 1.70 | 51 | 87 |
| 12 | 1.89 | 47 | 90 |
| 17, 20 | 1.20 | 63 | 76 |
| 18 | 1.99 | 45 | 91 |
| 21 | 1.25 | 62 | 78 |

[a]From **Table II.** [b]From equation (5) **Materials and methods.** [c]Calculated by $[1-0.3)^{1/m}]\times 100$. [d]Calculated by $[1-(0.3)^{m}]\times 100$.

### Discussion

A direct relationship between numbers of clonogenic cells plated and colonies counted is an important requirement for clonogenic assay systems. We have described a simple statistical method for testing whether colony formation is independent of cell concentration. Since the assumption that colony counts fit a Poisson distribution is crucial for this test, it was necessary to examine the dispersion of counts in replicate plates before it could be applied. Analysis of the results from 51 human tumour biopsy samples plated in our semi-solid culture system indicated that colony numbers are not linear with numbers of cells seeded for a significant proportion of samples. While the statistical test showed that some results did not fit the ideal relationship, actual inspection of the data suggested that two types of non-linearity were involved.

Our findings confirm and extend those of Meyskens *et al.* (1983), who have described in detail one type of non-linearity, that which we have termed type II, where cloning efficiencies are decreased at higher cell concentrations. One explanation for such behaviour by tumour cells (particularly cell lines and melanoma biopsy samples with high cloning efficiencies) is that the culture systems may be able to support only a limited volume of tumour growth (Thomson *et al.*, 1984). This mechanism is probably not a major factor in the cases of type II non-linearity reported here, since cultures were fed each week with fresh medium and relatively few colonies were evident at any cell dilution with many of the samples. In such cases, production of inhibitory factors by tumour cells, or by normal inflammatory cells plated together with tumour cells (Buick *et al.*, 1980; Hamburger *et al.*, 1983) may be involved.

Cloning efficiencies for about one-third of the samples examined appeared to increase with higher cell concentrations (type I). Curves of type I can be described by equations where cell concentrations is raised to a power greater than unity. Such relationships have the implication that more than one cell is required to give rise to one colony, thus supporting the use of the term colony forming unit (CFU) for human tumour colony assays. The size of the unit would be defined by the exponent of the relationship (slope, *m*, of the ln–ln transformed regression curve). In theory, if $m=2$, then two cells would be needed for formation of one colony. The cells involved might be two clonogenic cells or one clonogenic cell and one accessory cell such as a macrophage or lymphocyte (Hamburger *et al.*, 1983).

Cooperation between interacting cells could be modulated by cell-to-cell contact or by release of factor(s) into the medium. The latter possibility is supported by the finding that conditioned media from breast cancer cell lines will increase the cloning efficiencies of cells from freshly obtained breast tumour samples (Hug *et al*., 1984). It is attractive to speculate that samples with type I relationships have enhanced colony formation at high cell densities due to autocrine production of growth factors, possibly through activation of oncogenes (Waterfield *et al*., 1983; Doolittle *et al*., 1983). However, addition of non-specific nutrient factors to suboptimal media have been shown to affect the apparent linearity of colony formation by cells from human tumour cell lines (Eliason *et al*., 1984).

The two types of non-linearity have opposite consequences for chemosensitivity tests. As indicated by Meyskens *et al*. (1983) type II relationships can lead to predictions of drug resistance when, in fact, CFUs have been killed. This would provide an explanation for the false negatives (resistance to a drug *in vitro* with sensitivity of the tumour in the clinic) reported for ~10% of the cases in studies correlating *in vitro* assay results with clinical findings.

An even more important aspect with respect to the predictive potential of these assays is the rate of false positive results (i.e., sensitivity *in vitro* but resistance *in vivo*). They constitute nearly 30% of all predicted sensitivities (Salmon, 1984). Non-linear relationships of type I could provide an explanation for false positives since a moderate cell kill by a drug would result in a much larger reduction in colony numbers. Curves of this type can be fitted using a ln–ln transformation as described by equation (5). It must be pointed out that the values for *m* calculated by standard regression analysis are only a rough approximation because the variances are not equal. More accurate estimates can be obtained by using weighted values for the regression to equalize the variances. In Table VI the ln–ln regression equations for each ovarian sample from Table II with *m* greater than one have been used to calculate the reduction in cells when colony numbers are reduced by 70% (Salmon *et al*., 1981) and conversely the reduction in colony numbers that represents a cell kill equal to 70%. Obviously, calculations of this type could be of potential benefit for improving the predictive accuracy of clonogenic assays. Since the logarithmic transformation can be applied to linear relationships as well as type I non-linearity, this approach could be used with about 80% of the samples in our series.

It may be argued that the high incidence of non-linearity we have observed is inherent to our culture system, which employs a single layer of methylcellulose rather than a double layer of agar and contains a relatively low concentration of serum (5%). However, reported non-linearity of colony formation in a more conventional double layer agar system (Meyskens *et al*., 1983) suggests it to be a more universal phenomenon and a property of the samples themselves.

In light of the cellular heterogeneity of tumour samples, both in terms of biological characteristics of the tumour cells and with respect to the content of other non-malignant cells, it would be advisable to include as internal controls plates with appropriate cell dilutions for every sample cultured. Detailed knowledge of the relationship between numbers of colonies and cells seeded in each assay could provide a means for correcting potential artifacts in chemosensitivity tests. Furthermore, the Poisson distribution of progenitor cells in replicate plates has important consequences for statistical treatment of results from clonogenic assays.

The authors thank I. Sénéchaud, V. Roosens and D. Petral for technical assistance, Drs B. Mermillod and N. Odartchenko for critical reading of the manuscript and Drs P. Alberto and F. Krauer for their guidance.

This work was supported by grants from the Swiss National Science Foundation, Swiss League Against Cancer, Geneva League Against Cancer, Fonds de Recherche sur les Lymphomes Malins and Fondazione San Salvatore.

### References

ARMITAGE, P. (1971). *Statistical Methods in Medical Research*. Blackwell: Oxford.

BUICK, R.N., FRY, S.E. & SALMON, S.E. (1980). Effect of host-cell interactions on clonogenic carcinoma cells in human malignant effusions. *Br. J. Cancer*, **41**, 695.

DIEM & LENTER (Eds). (1980). *Scientific Tables*. 7th edition, Ciba-Geigy, Basel.

DOOLITTLE, R.F., HUNKAPILLER, M.W., HOOD, L.E. & 4 others (1983). Simian sarcoma virus *onc* gene, v-sis, is derived from the gene (or genes) encoding a platelet-dervied growth factor. *Science*, **221**, 275.

ELIASON, J.F. (1984). Long-term production of hemopoietic progenitors in cultures containing low levels of serum. *Exp. Hematol.*, **12**, 559.

ELIASON, J.F., FEKETE, A. & ODARTCHENKO, N. (1984). Improving techniques for clonogenic assays. *Recent Results Cancer Res.*, **94**, 267.

ENGH, G.J. VAN DEN (1976). *Early Events in Haemopoietic Cell Differentiation*. Thesis, University of Leiden.

HAMBURGER, A.W., WHITE, C.P. & DUNN, F.E. (1983). Modulaion of tumor colony growth by irradiated accessory cells. *Br. J. Cancer*, **48**, 675.

HENDRY, J.H. (1973). The effect of spleen colony production of the death of recipient mice. *Cell Tissue Kinet.*, **6**, 97.

HUG, V., HAYNES, M., RASHID, R., SPITZER, G., BLUMENSCHEIN, G. & HORTOBAGYI, G. (1984). Improved culture conditions for clonogenic growth of primary human breast tumours. *Br. J. Cancer*, **50**, 207.

ISCOVE, N.N., GUILBERT, L.J. & WEYMAN, C. (1980). Complete replacement of serum in primary cultures of erythropoietin-dependent red cell precursors (CFU-E) by albumin, transferrin, iron, unsaturated fatty acid, lecithin and cholesterol. *Exp. Cell Res.*, **126**, 121.

MEYSKENS, F.L., Jr., THOMSON, S.P., HICKIE, R.A. & SIPES, N.J. (1983). Potential biological explanation of stimulation of colony growth in semi-solid agar by cytotoxic agents. *Br. J. Cancer*, **48**, 863.

MEYSKENS, F.L., Jr., THOMSON, S.P. & MOON, T.E. (1984). Quantitation of the number of cells within tumor colonies in semi-solid medium and their growth as oblate spheroids. *Cancer Res.*, **44**, 271.

SALMON, S.E., HAMBURGER, A.W., SOEHNLEN, B., DURIE, B.G.M., ALBERTS, D.S. & MOON, T.E. (1978). Quantitation of differential sensitivity of human-tumor stem cells to anticancer drugs. *N. Engl. J. Med.*, **298**, 1321.

SALMON, S.E., MEYSKENS, F.L., Jr., ALBERTS, D.S., SOEHNLEN, B. & YOUNG, L. (1981). New drugs in ovarian cancer and malignant melanoma: *in vitro* phase II screening with the human tumor stem cell assay. *Cancer Treat. Rep.*, **65**, 1.

SALMON, S.E. (1984). Human tumor colony assay and chemosensitivity testing. *Cancer Treat. Rep.*, **6**, 117.

SELBY, P., BUICK, R.N. & TANNOCK, I. (1983). A critical appraisal of the 'human tumor stem-cell assay'. *N. Engl. J. Med.*, **308**, 129.

SLOCUM, H.K., PAVELIC, Z.P., RUSTUM, Y.M. & 4 others (1981). Characterization of cells obtained by mechanical and enzymatic means from human melanoma, sarcoma and lung tumors. *Cancer Res.*, **41**, 1428.

THOMSON, S.P., MOON, T.E. & MEYSKENS, F.L., Jr. (1984). Kinetics of clonogenic melanoma cell proliferation and the limits on growth within a bilayer agar system. *J. Cell. Physiol.*, **121**, 114.

WATERFIELD, M.D., SCRACE, G.T., WITTLE, N. & 7 others (1983). Platelet-derived growth factor is structurally related to the putative transforming protein $P28^{sis}$ of simian sarcoma virus. *Nature*, **304**, 35.